# Balanced Dopamine Is Critical for Pattern Completion during Associative Memory Recall

**DOI:** 10.1371/journal.pone.0015401

**Published:** 2010-10-27

**Authors:** Fei Li, L. Phillip Wang, Xiaoming Shen, Joe Z. Tsien

**Affiliations:** 1 Department of Developmental and Behavioral Pediatrics of Shanghai Children's Medical Center & Shanghai Key Laboratory of Children's Environmental Health, XinHua Hospital, Shanghai Jiao Tong University School of Medicine, Shanghai, China; 2 Brain and Behavior Discovery Institute, Medical College of Georgia, Augusta, Georgia, United States of America; Université Pierre et Marie Curie, France

## Abstract

Pattern completion, the ability to retrieve complete memories initiated by partial cues, is a critical feature of the memory process. However, little is known regarding the molecular and cellular mechanisms underlying this process. To study the role of dopamine in memory recall, we have analyzed dopamine transporter heterozygous knockout mice (DAT^+/−^), and found that while these mice possess normal learning, consolidation, and memory recall under full cue conditions, they exhibit specific deficits in pattern completion under partial cue condition. This form of memory recall deficit in the dopamine transporter heterozygous knockout mice can be reversed by a low dose of the dopamine antagonist haloperidol, further confirming that the inability to retrieve memory patterns is a result of dopamine imbalance. Therefore, our results reveal that a delicate control of the brain's dopamine level is critical for pattern completion during associative memory recall.

## Introduction

Memory recall involves a recapitulation of previously acquired information [1], [2]. Depending on the state of recall conditions, memory retrieval can occur with most or all of the previously encountered cues associated with learning (e.g. seeing a person and hearing his voice simultaneously, or revisiting one's hometown that did not change much, etc). On the other hand, in many cases, memory retrievals usually take place when only subsets of initial cues are present (e.g. reconstructing the old street maps of one's hometown when only a few old landmarks remained unchanged). This is known as pattern completion in which the brain reconstructs and retrieves entire memory patterns from partial external cues or self-initiated internal processes. Currently, little is known about the actual molecular and cellular mechanisms underlying the pattern completion of memory recall. However, emerging studies indicate that monoamine signaling may play a role in memory retrieval [3].

In this study, we set out to examine how the modulatory neurotransmitter dopamine plays a role in regulating memory pattern completion during partial cue recall. Dopamine is a key neurotransmitter that can influence cognition, emotion, and movement. Abnormal dopaminergic transmission has been implicated in a number of psychiatric and neurological disorders including attention deficit and hyperactivity disorder (ADHD), Schizophrenia, and Parkinson's disease [4]–[8]. Although dopaminergic neurons originate only from the ventral tegmental area and substantia nigra compacta, their outputs project to almost everywhere in the brain, including the prefrontal cortex, medial temporal lobe, and hippocampus, regions known to be activated during memory retrieval [3], [9]–[14]. It also should be noted that dopamine was thought functionally crucial for attention and working memory mediated by above brain regions [15]–[18], both of which were implied in the process of memory retrieval under partial cue conditions [19]. As the primary cellular mechanism to terminate dopamine signaling, the dopamine transporter (DAT), located at the neuronal presynaptic terminals, reuptakes dopamine from the synaptic cleft back into the dopaminergic neurons. As such, DAT is a critical molecule in regulating synaptic levels of dopamine, and consequently determining the temporal duration of dopamine actions on the local neural circuits. Indeed, genetic knockout of the dopamine transporter gene results in profound impairments. The homozygous DAT-KO mice suffer from overt abnormalities including growth retardation, robust locomotor hyperactivity, and many other impairments including deficits in habituation and social interaction as well as impaired gut motility, respiratory control, etc. [6], [20], [21]. The overall defects in the homozygous DAT-KO mice have made it less suitable to probe the role of dopamine in regulating memory processes.

Interestingly, the heterozygous knockout mice (DAT^+/−^ mice), still possessing an allele of the functional DAT gene, seem to be quite normal in their overall gross behaviors [6], [20], [21]. Thus, the DAT^+/−^ mice may provide a valuable model for studying some of the delicate, but important phenotypes, such as associative memory processes, and related mechanisms regulated by dopaminergic circuitry. Here, we used a set of behavioral paradigms to assess the functional consequences of dopamine imbalance on pattern completion during associative memory recall.

## Results

**Figure 1:**
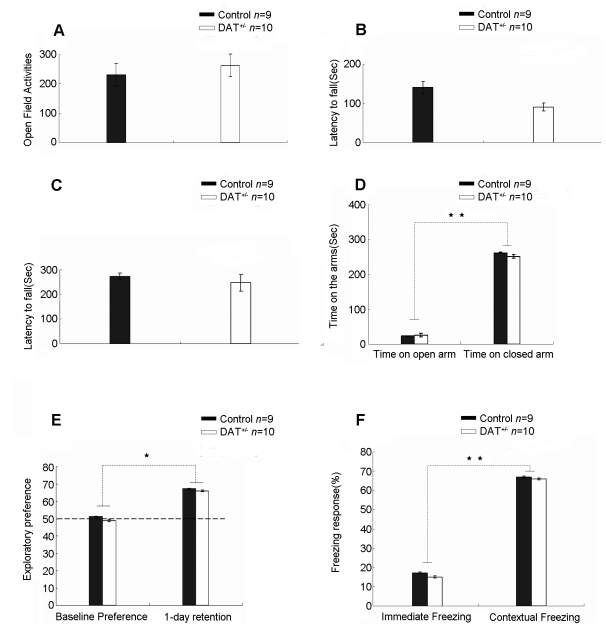
Normal performance of DAT^+/−^ mice in basic behaviors. (A) Normal open field locomotor behavior in DAT^+/−^ mice (p>0.05). (B) Indistinguishable motor learning in the 1-hour rotarod tests between the DAT^+/−^ mice and wild-type littermates (p>0.05). (C) Normal performances of the DAT^+/−^ mice in the 1-day rotarod tests (p>0.05). (D) Indistinguishable performances on the elevated plus maze, suggesting no changes in their anxiety level in DAT^+/−^ mice (p>0.05). (E) DAT^+/−^ mice showing normal learning and memory in the novel object recognition test. 1-day retention tests were used. (p>0.05). (F) DAT^+/−^ mice showing normal hippocampal dependent contextual emotional memory as assessed by contextual fear conditioning. 1-day retention tests were used (p>0.05). Control mice, n = 9; KO mice, n = 10. Data were calculated as Mean ± SEM. Either ** or *Indicates a significant difference between groups for a given point in time (**p<0.01, *p<0.05). Either ^★★^or ^★^Indicates a significant difference within groups for a given point in time (^★★^p<0.01, ^★^p<0.05).

To investigate the role of dopamine in memory retrieval, we used the heterozygous dopamine transporter knockout mice (DAT^+/−^). We employed a battery of basic behavioral measurements to assess their open field locomotor activity (Figure 1A), rotarod performances (Figure 1B and 1C), and found that these heterozygous knockout mice are completely normal. We also confirmed that the DAT^+/−^ mice exhibit indistinguishable performances in the anxiety level as measured by the elevated plus maze (Figure 1D).

In addition, we assessed the basic learning and memory functions in the DAT^+/−^ mice. Firstly, we used the novel object recognition test and observed that these mice displayed completely normal behavioral performances in the 1 day retention tests as compared to their wild-type littermate control (Figure 1E). Moreover, these mice also exhibit normal 1-day fear conditioned retention that is indistinguishable to their wild-type control mice (Figure 1F). Therefore, these results suggest that the DAT^+/−^ mice have normal learning and memory function in these two forms of primary memory tests.

**Figure 2:**
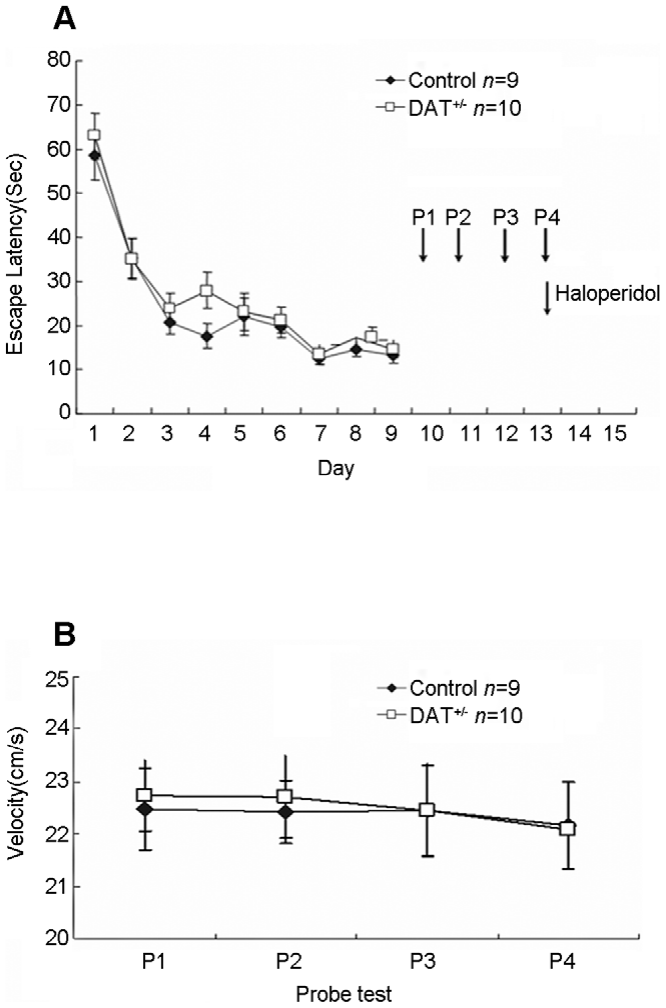
Normal acquisition and consolidation of spatial reference memory in DAT^+/−^ knockout mice without velocity difference. (A)Normal acquisition of spatial reference memory in DAT^+/−^ mice (n = 10) and their control littermates (n = 9) as measured by escape latency (p>0.05). Four probe tests (P1, P2, P3, and P4) were conducted. (B) Indistinguishable swimming speed in DAT^+/−^ mice (n = 10) and their control littermates (n = 9) (p>0.05). Data were calculated as Mean ± SEM. Either** or *Indicates a significant between-group difference for a given time point or probe test (**p<0.01, *p<0.05). Either ^★★^or ^★^Indicates a significant within-group difference for a given probe test (^★★^p<0.01, ^★^p<0.05).

The spatial reference memory test has been previously used to assess the pattern completion of memory recall. We subjected the DAT^+/−^ mice and wild-type controls to this task. Using the spatial reference memory protocol that was described previously [22], we trained these mice in the hidden-platform water maze. The training consisted of four trials per day, with a one hour-interval between trials. We found that both the DAT^+/−^ mice and the wild-type mice displayed comparable learning and memory consolidation over the course of 10 day sessions and with similar swimming speeds (Figure 2A and 2B).

**Figure 3:**
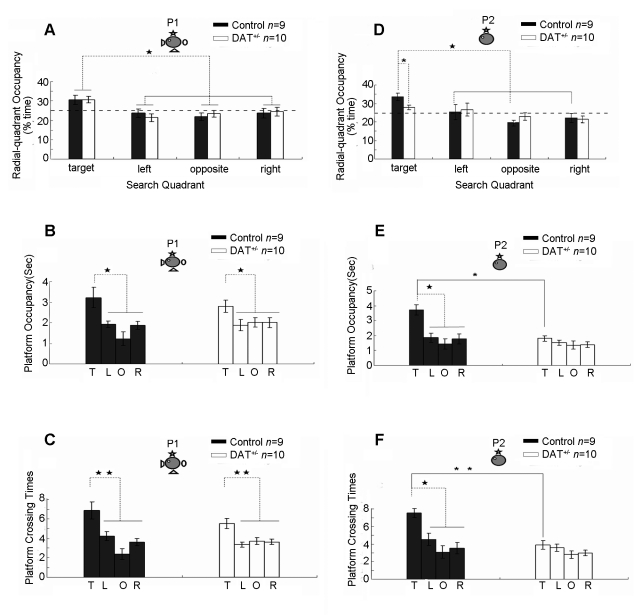
Selective deficits in pattern completion during retrieving spatial reference memory in DAT^+/−^ knockout mice. (A). Both DAT^+/−^ mice and control mice exhibited strong preference to the target quadrant where the platform was previously located under full cue conditions. The location of the hidden-platform and four visual cues on the surrounding black curtain wall are illustrated. (B) Normal retrieval in DAT^+/−^ mice under full-cue conditions were confirmed by the measurement of platform occupancy. (C) The measurement of platform crossings further shows that both types of mice crossed the phantom platform location more significantly than the similar locations in other quadrants. (D) Impairment of memory recall in DAT^+/−^ mice under partial cue conditions as indicated by lack of preference to the target quadrant on P2 trial. (E) Impaired pattern completion in DAT^+/−^ mice as indicated by chance level in platform occupancy. (F) Impaired pattern completion in DAT^+/−^ mice as indicated by chance level in platform crossings. T: Target quadrant, L: Left quadrant; O: Opposite quadrant; R: Right quadrant. Either** or *Indicates a significant between-group difference for a given time-point or probe test (**p<0.01, *p<0.05). Either ^★★^or ^★^Indicates a significant within-group difference for a given probe test (^★★^p<0.01, ^★^p<0.05).

Next, we examined their memories of the hidden platform location by using the probe test (P1) on day 11, one day after the completion of the last training session. As measured by quadrant occupancy, both DAT^+/−^ mice and their control littermates were able to focus their search in the target quadrant in the presence of full cues (Figure 3A). Moreover, DAT^+/−^ mice also exhibited a strong preference in the phantom platform area, and there was no difference in comparison to the controls' platform occupancy (Figure 3B). Furthermore, as expected, both DAT^+/−^ mice and the wild-type littermates exhibited a significant increase in the number of crossings (Figure 3C). Thus, all of these measurements suggest that DAT^+/−^ mice can learn this task normally and retrieve this associative memory normally under full-cue conditions.

To determine whether the delicate balance of dopamine is essential for pattern completion under partial cue conditions, we conducted the second probe test (P2) the next day by removing three of the four distal cues (day 12). To avoid any possible extinction from the previous recall session, one more block (4 trials) of training was delivered 1 hour after P1 probe test. During this partial-cue probe trial, while the control mice continued to concentrate their search time in the target quadrant rather than the other quadrants, the DAT^+/−^ mice showed only chance-level performance as measured by target quadrant occupancy (Figure 3D). Moreover, the measurement of occupancy of the phantom platform areas further confirmed that these DAT^+/−^ mice were impaired in remembering the platform location (Figure 3E). This retrieval deficit was also shown by the lack of an increase in the number of platform crossings (Figure 3F), whereas the wild-type littermates were fully capable of performing partial-cue memory recall. Therefore, these data suggest that the DAT^+/−^ mice are deficient in retrieving spatial reference memories under partial-cue conditions.

**Figure 4:**
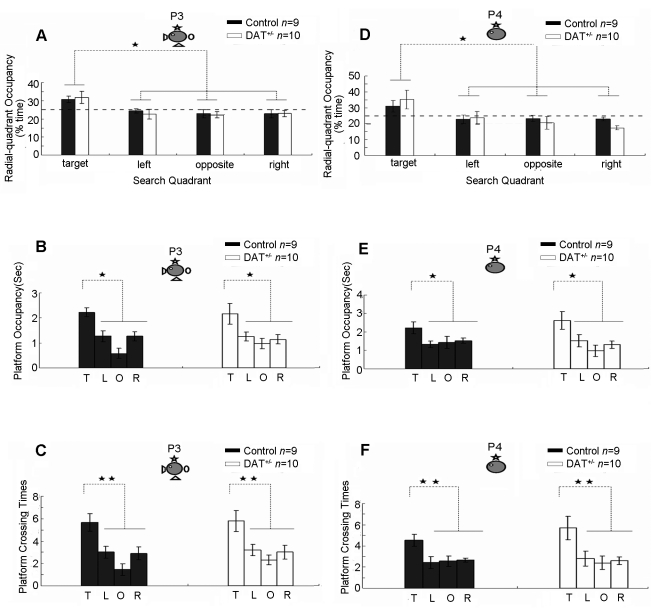
Reversal of pattern completion deficits in DAT^+/−^ mice using haloperiodol after P1 and P2 probe tests. Normal retrieval in DAT^+/−^ mice under full cue conditions (P3 trials) as measured by target quadrant occupancy (A), platform occupancy (B), and platform crossings (C). No drug was given during this full cue condition test. These mice received two additional trainings on days 13 and 14 (see Figure 1A for probe trial regimen). On the fourth probe trial (P4), DAT^+/−^ mice received systemic injection of a low dose of haloperidol 30 minutes prior the recall tests and the wild-type littermates received a saline injection as a control. Their ability in pattern completion during memory recall was measured by quadrant occupancy (D), platform occupancy (E), and platform crossings (F).These measurements show that DAT+/− mice now can perform as well as the wild-type mice. T: Target quadrant, L: Left quadrant; O: Opposite quadrant; R: Right quadrant. The location of the hidden-platform and one remaining cue hung on the surrounding black curtain wall are illustrated. Either** or *Indicates a significant between-group difference for a given time-point or probe test (**p<0.01, *p<0.05). Either ^★★^or ^★^Indicates a significant within-group difference for a given probe test (^★★^p<0.01, ^★^p<0.05).

Finally, we asked whether we could restore pattern completion in these DAT^+/−^ mice using pharmacological methods. It has been reported that a low dose of the dopamine antagonist, haloperidol, could be useful in relieving certain dopamine disorders [20]. The rationale is that the low dose of haloperidol may be able to somewhat dampen the effect of the elevated dopamine in the heterozygous mice which have insufficient dopamine reuptake due to the loss of one allele of the normal dopamine transporter gene. We applied the same set of mice to the rescue experiment. On day 13 and day 14, we subjected the above mice to the third probe trial (P3) under full-cue conditions and the fourth probe trial (P4) under partial-cue conditions. Again, in order to counteract any extinction that may have occurred during the probe trial, we conducted one more block (4 trials) of training 1 hour after completion of either P2 or P3 probe test. Our measurements of target quadrant occupancy on the P3 probe test shows that both the DAT^+/−^ mice and the control littermates concentrated their search in the target quadrant in the presence of full cues (Figure 4A). Furthermore, their normal memory recall was again evidenced by the measurement of platform occupancy (Figure 4B) as well as the number of platform crossings (Figure 4C). Thus, these mutant mice were fully capable of retrieving spatial memory under full-cue conditions.

On the day 14, we removed three of the four distal cues and conducted the fourth probe trials (P4) under the partial-cue condition. We injected the DAT^+/−^ mice intraperitoneally with a low dose of haloperidol (0.002 mg/kg of body weight) 30 minutes before the retention tests. The wild-type littermates received a saline injection as a control. We found that the DAT^+/−^ mice concentrated their search time in the target quadrant and showed statistically similar performances in comparison to the wild-type counterparts (Figure 4E). Also, the measurement of occupancy of the phantom platform areas further substantiated that these DAT^+/−^ mice can recall the platform location (Figure 4F). Their normal memory recall was again confirmed by an increase in the number of platform crossings, which was at the same level as that of the wild-type mice (Figure 4G). Thus, these experiments suggest that pattern completion deficits originally observed in the DAT^+/−^ mice may be caused by the dopamine imbalance.

**Figure 5:**
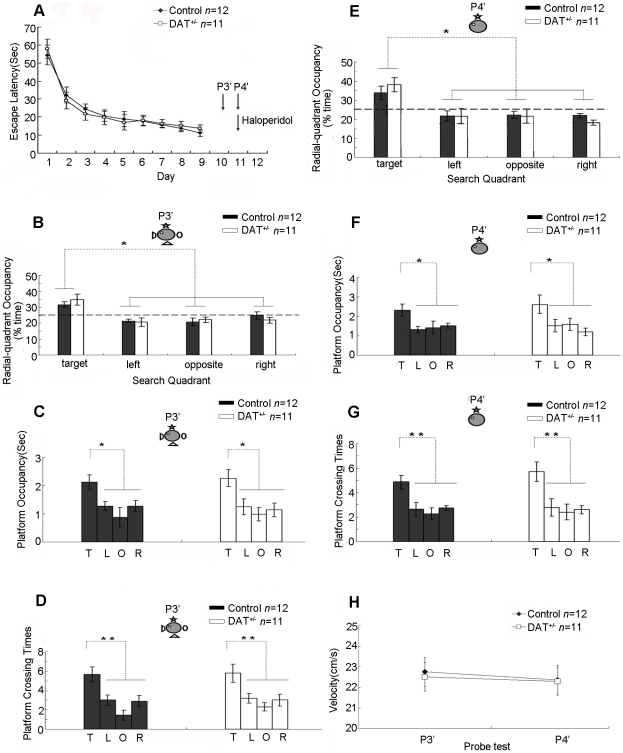
Rescue of pattern completion deficits using haloperiodol in DAT^+/−^ mice which did not receive multiple probe tests. (A) Normal acquisition of spatial reference memory in two other groups of DAT^+/−^ mice and their control littermates as measured by escape latency. Two other probe tests (P3' and P4') were conducted right after training section. Normal retrieval in DAT^+/−^ mice under full cue condition as measured by target quadrant occupancy (B), platform occupancy (C), and platform crossings (D). No drug was given during this full cue condition test (P1). These mice received an additional block (4 trials) of training one hour after the day 10 full cue probe trial (see Figure 1C for probe trial regimen). On the partial cue trial (P2), DAT^+/−^ mice received systemic injection of a low dose of haloperidol 30 minutes prior the recall tests and the wild-type littermates received a saline injection as a control. Their ability in pattern completion during memory recall was measured by quadrant occupancy (E), platform occupancy (F), and platform crossings (G). These measurements show that DAT^+/−^ mice are now able to perform as well as the wild-type mice. (H) Indistinguishable swimming speed in DAT^+/−^ mice and the control littermates. T: Target quadrant, L: Left quadrant; O: Opposite quadrant; R: Right quadrant. The location of the hidden platform and one remaining cue hung on the surrounding black curtain wall are illustrated. Either** or *Indicates a significant between-group difference for a given point in time or probe test (**p<0.01, *p<0.05). Either ^★★^or ^★^Indicates a significant within-group difference for a given probe test (^★★^p<0.01, ^★^p<0.05).

In order to exclude the possibility that the results for the phenotype injected with haloperidol in P4 probe trial was due to overtraining during the repeated probe tests, we used another set of DAT^+/−^ and control littermates and repeated the entire experiment. As expected, both DAT^+/−^ mice and their wild-type mice displayed good learning rates over the course of the 10 day training sessions (Figure 5A). On day 11, we then subjected these mice to full-cue recall tests, there is no significant difference in memory retention test results between the DAT^+/−^ mice and the control littermates as measured by quadrant occupancy (Figure 5B), target quadrant occupancy (Figure 5C), and the number of platform crossings (Figure 5D). One hour after the completion of the full-cue probe test, we retrained these mice with one more block of training to prevent any extinction effect. On day 12, these mice were subjected to the partial-cue recall tests. A low dose of haloperidol (or saline) for the controls was injected into the mice intraperitoneally 30 minutes before the partial-cue trial. We found that this treatment has indeed resulted in the normal performances in the mutant mice. The mutant and control mice exhibited comparable performances in quadrant occupancy (Figure 5E), target quadrant occupancy (Figure 5F), and the number of platform crossings (Figure 5G). The measurement of their swimming speeds also revealed no differences (Figure 5H). Therefore, these data clearly demonstrated that the rescued partial-cue retrieval deficit in the DAT^+/−^ mice by haloperidol was not due to repeated overtraining during multiple probe trials.

## Discussion

While the dopamine system is well known to be crucial for the regulation of many cognitive processes [8], [16]–[18], [23]–[25], our present study provides evidence for the first time that dopamine imbalance, resulting from the loss of one allele of the normal dopamine transporter gene, caused a specific deficit in pattern completion during associative spatial memory recall. This memory recall deficit is evident only under the partial-spatial-cue conditions, but not under the full-cue conditions. Moreover, this memory recall deficit seems to reflect a highly specific form of memory deficit because broad aspects of basic behaviors (open-field locomotion, rotarod, and anxiety) and other forms of memory such as contextual fear conditioning and novel object recognition remain normal.

There are several potential molecules and cellular scenarios that may collectively contribute to the observed spatial partial-cue-triggered recall deficit, among which dopamine is thought to be a major candidate molecule underlying this memory process this is because attention and working memory, primarily controlled by dopamine signals, are reported to be critical for the retrieval of spatial memories [15]–[18], [26]. It is well known that dopaminergic neurons, originating only from the ventral tegmental area and substantia nigra compacta, project to almost everywhere in the brain, including the prefrontal cortex, medial temporal lobe, and hippocampus [5], [19], [27]–[28], regions known to be activated during memory retrieval as well as attention processes [3], [9]–[14], [29], [30]. Given the broad evidence that dopamine is essential for attention and working memory [15]–[18] and that the genetic polymorphism in the DAT gene is thought to be implicated in ADHD [31]–[33], it is possible that both attention and working memory might play a role in pattern completion of memory retrieval under the partial-cue conditions through DAT-mediated dopamine regulation. Thus, memory pattern completion deficits observed in the DAT heterozygous mutant mice may be due to the mouse's inability to meet the increased attentional demands during partial cue-based memory recall as a result of synaptic dopamine disturbance.

Our finding that dopamine imbalance resulted in memory retrieval deficit is also interesting in light of the clinical dementia observed in Parkinson's patients. These patients usually seem to retain the ability to learn, consolidate and store new memory but are profoundly impaired in retrieving memories especially under partial external cues or self-initiated recall [34], [35]. This deficit is especially profound when explicit cues were absent [8], [23], [34]–[36], thereby further indicating that dopamine might be involved in the memory recall process. These types of deficits in memory recall in Parkinson's patients are in stark contrast to the memory deficits in other neurotransmitter systems [37] or the early dementia in Alzheimer's patients who are typically impaired in learning and consolidating new memories while preserving the ability to recall old memories [34], [35]. This illustrates the need to develop different therapeutic strategies because of the different vulnerabilities to distinct molecular and temporal processes within memory circuitries.

Our demonstration that pattern completion can be completely rescued by injection of haloperiodol at the time of recall reinforces the idea about the role of balanced dopamine levels in memory retrieval. This pharmacological rescue experiment provides additional evidence for temporal specificity that causes the partial cue-based recall deficit. It should be noted that dopamine dysfunctions in DAT^+/−^ mice and in Parkinson patients are quite different from each other, yet both leads to pattern retrieval deficits. This commonality provides collective support for the notion that the delicate balance of the dopamine system is crucial for memory retrieval, and imbalance in either direction (up or down) would cause deficits in memory pattern completion during recall. Importantly, we would like to point out that our present analysis should not be interpreted as evidence for using the DAT mutant mice as a Parkinson's disease model. On the other hand, *in vivo* measurement of dopamine in DAT homozygous knockout mice shows a significant reduction in dopamine release triggered by burst stimulation [38]–[40]. This indicates that the ability to translate the neural activity burst into dopamine signals in the various brain regions of knockout mice may be deficient. It is conceivable that reduced dopamine ratio changes can lead to altered physiological changes in the firing patterns within the neural circuits involved in memory processing. Currently, it is not known whether the similar alteration also occurs in the DAT heterozygous knockout mice or in Parkinson's disease patients.

Although little is known regarding the neural circuits activated during spatial memory recall, it is likely that it recruits multiple regions including the prefrontal cortex, the medial temporal cortex, and the hippocampus. This fits well with the anatomical evidence that the dopaminergic outputs from the ventral tegmental area projects heavily to the ventral CA1 area and the entorhinal cortex[13], [28]. This prefrontal-hippocampal-VTA loop may play a crucial role for generating contextual familiarity which, in turn, promotes pattern completion during partial cue-based spatial memory recall through facilitation of dopamine-regulated attention [3], [14], [26], [28]. It will be important in future studies to further define the anatomical loci from which the observed pattern completion deficits originate. It would be especially interesting to investigate candidate sites such as the anterior cingular cortex, the temporal cortex and the hippocampus using pharmacological, genetic, and large-scale in vivo recording techniques [11], [41]–[44]. It is also important to assess whether genetic compensation or slow changes in the mutant brain contribute to the observed recall deficits. There are also indications that other neurotransmitter systems may also be critically involved in the regulation of memory retrieval [3], [37], [45], [46], and it would be highly interesting to examine and compare their dynamic interactions between the partial cue-triggered pattern completion and the full cue-based memory retrieval. In conclusion, our study suggests that a delicate balance in dopamine levels is crucial for pattern completion during associative spatial memory recall.

## Materials and Methods

### Ethic statements

All animal work described in the study have been conducted according to NIH guidelines and approved by Institutional IACUC committee at Medical College of Georgia (Approval AUP number: BR07-11-001).

### Production and Genotyping of Mutant Mice

The DAT mice were a generous gift from the laboratory of Dr. XiaoXi Zhuang of the University of Chicago. Breeding and genotyping of DAT heterozygous knockout mice are the same as described [6]. For our experiments, both male and female mice were equally used at a ratio of 1∶1. PCR for DAT^+/−^ mice was followed by protocol as described [6]. All mice were maintained under standard conditions (23.1°C, 50.5% humidity) in the Animal Facility of the Medical College of Georgia. All experiments were conducted in a soundproofed and specialized behavior room. All experimenters were blind to the genotype of the individual animal.

### Novel-Object Recognition Task

The experimental protocol was the same as described previously [37], [47]. Briefly, mice were individually habituated to an open-field box (20×20×10 high inches) for 3 days. During the training sessions, two novel objects were placed in the open field, and the animal was allowed to explore for 15 min. The time spent exploring each objects was recorded. During the one-hour recall tests, the animal was placed back into the same box, in which one of the familiar objects during training was replaced by a novel object, and allowed to explore freely for 15 min. A preference index, a ratio of the time spent exploring any one of the two objects (training session) or the novel one (retention session) over the total time spent exploring both objects, was used to measure recognition memory.

### Open Field and Rota-rod Tests

The protocols were the same as described [48]. For the measurement of the open field activity, mice were placed in an open field, made of a 14×14 inch black box. The box was marked by 2×2 inch small square grids (7 squares by 7 squares with 49 squares in total). The open field activity of animals was measured by the number of crosses that the mice have passed within the 3-minute period. For the measurement of Rota-rod test, the mice were placed to an accelerating rotating wood-rod. The rod is 12 inched long and 1 inch in diameter. The initial rotation speed was at 4 rpm and then steadily accelerated to 40 rpm. The performance was measured by the amount of time (in seconds) that mice managed to remain on the rotating rod during either the five-minute or the one-hour recall tests.

### Elevated Plus Maze Tests

The protocols were the same as described [49]. The elevated plus maze is made of stainless steel, which is painted matte black, and consists of four arms (two open without walls and two enclosed by 15.25 cm high walls) 30 cm long and 5 cm wide. Each arm of the maze is attached to sturdy metal legs such that it is elevated 40 cm above the table on which it rests. Activity was recorded by a digital camera (Logitech Camera, Model No. N231) placed 130 cm above the maze. Testing took place under dim light (one 40-W and one 60-W soft white light bulb, both angled to create indirect lighting on the maze) during the light phase of the circadian cycle (between 0900 h and 1400 h). The maze was cleaned with 5% acetic acid between tests. White noise (30 dB) masked extraneous background noise. On the test day, animals were brought into the testing room in their home cages, and each pair of animals was then removed from its home cage and placed in a separate holding cage for 5 min before being placed on the maze. Animals were placed individually in the center of the maze, with head position counterbalanced between mice, and behavior was recorded for 5 min. the time spent on the open arm and closed arm (when all four paws of the rodent are on the open or closed arm) were recorded and analyzed.

### Contextural Fear Conditioning

Fear-conditioning was performed as previously described [45]. The experiment was carried out in a fear-conditioning system, a chamber situated in a sound attenuated box with a house light on the ceiling and a stainless steel grid floor (Coulbourn Instruments, Whitehall, PA). The grid floor was wired to a shock generator and an auditory signal originated from a loudspeaker attached on the wall of the chamber. All stimuli were controlled automatically using a personal computer with a Graphic State program. A video camera was placed in front of the cage to record behavior. Mice were handled for 3 days and then habituated to the training chamber for 5 min. The conditioned stimulus (CS) used was an 85 dB sound at 2.8 kHz, while the unconditioned stimulus (US) was a continuous scrambled foot shock at 0.8 mA for 2 s. After a single co-terminating CS/US paring, the animal remained in the chamber for another 30 s for the measurement of immediate freezing. During the retention test, each mouse was placed back into the same chamber, and the freezing responses were recorded for 5 min (contextual freezing response). All tests were videotaped under red light. Total freezing time was measured as an index of fear memory. Freezing behavior was defined as a complete lack of movement excluding respiration. Freezing behavior was scored by software (Coulbourn Instruments) and converted to freezing response [freezing response  =  (total freezing time/total testing time) ×100%].

### Spatial Reference Memory Tests

The spatial reference memory test was the hidden-platform water maze. We followed the protocol as described previously by Nakazawa et al. [22]. The training consisted of four trials per day, with one hour between trials. The movement of mice was tracked by video camera and measured by software (Noldul Information Technology, Netherlands). The escape latency to the platform as well as quadrant occupancy and platform crossing were all recorded and analyzed. The pool has a diameter of 118 cm and the platform is 9.5 cm in diameter. Four probe tests were performed. The first probe test (P1) was conducted the day following the last training session under full-cue conditions (Day 11). The second probe test (P2) was conducted on day 12 under partial-cue conditions (by removing three of the four visual cues hung on the black curtain wall). For the DAT^+/−^ mice, we performed the third probe trial (P3) on day 13 under full-cue conditions and fourth probe trial (P4) on day 14 under partial-cue conditions. One more block (4 trials) of training was delivered 1 hour after P1, P2 and P3 probe tests respectively, in order to counteract any extinction that may have occurred during the probe trial. Furthermore, in order to exclude the compounding effect of likely overtraining before P4 (probe test with partial-cue and haloperidol injection), we subjected another group of DAT^+/−^ mice as well as their control wild-type littermates to two additional probe tests (P3' and P4' trials). The P3' probe test was conducted one day after the last training session under full-cue conditions (day 10). The P4' probe test was conducted on day 11 under partial-cue conditions. During all of our probe tests, the platform was removed and the mice were allowed to swim in the pool for the same amount of time as used during training (60 sec). The time spent in each quadrant was recorded. To restore the dopamine levels [6], [20], [21], mice from DAT^+/−^ and control groups were all injected intraperitoneally with either haloperidol (0.002 mg/kg of body weight) or with saline 30 minutes before the P4 and P4' probe trials.

### Data Analysis

To account for intra-animal correlations between repeated measurements, linear mixed models were employed to estimate the behavioral performance in the Morris water maze, novel object recognition, contextural fear conditioning and rota-rod tests. The Tukey–Kramer method was used to determine the significance of those behavioral measurements between DAT^+/−^ mice and the control littermates. In the open field and elevated plus maze tests, One-way ANOVA and post hoc Dunnett's test were used to determine genotype effects. Continuous variables are presented as the mean and standard error of the mean (SEM). Data were analyzed using SPSS version 13.0 (SPSS Inc.,Chicago, IL). Differences were considered significant when P<0.05.
